# The preference of *Trichopria drosophilae* for pupae of *Drosophila suzukii* is independent of host size

**DOI:** 10.1038/s41598-020-80355-5

**Published:** 2021-01-13

**Authors:** Benedikt J. M. Häussling, Judith Lienenlüke, Johannes Stökl

**Affiliations:** 1 Department of Evolutionary Animal Ecology, Bayreuth University, Bayreuth, Germany; 2 Institute for Insect Biotechnology, Justus-Liebig University, Giessen, Germany

**Keywords:** Invasive species, Agroecology, Entomology

## Abstract

Controlling the cosmopolitan pest *Drosophila suzukii* (spotted wing drosophila) is a challenge for fruit growers. A promising agent for biological control of that pest are parasitoid wasps. Especially the widespread pupal parasitoid *Trichopria drosophilae* had shown the ability to parasitise the pest fly. However, as a biocontrol agent, parasitoids can only be effective when they prefer the pest to other insects. Until now studies have been inconsistent concerning the preference of *T.* *drosophilae* for *D. suzukii* and whether the preference depends on pupal volume. To clarify this inconsistency, we used video recordings of parasitisation experiments with a set up to observe the direct host preference of the parasitoid. Additionally, the volume of each host pupa was measured. We found significant preference of *T. drosophilae* for *D. suzukii* pupae independent of the pupal size and of the host species the wasps were reared on. The article also discusses the sex ratio and the success of the parasitoid in the different pupae characteristics.

## Introduction

The range and speed of the distribution of invasive insect pest species are increasing with globalisation across all agricultural ecosystems. These insects can bring considerable negative impacts along with potential massive economic losses for farmers^1,2^. An example par excellence is the invasive pest *Drosophila suzukii* Matsumura (Diptera: Drosophilidae), also called the spotted wing drosophila (SWD). SWD is endemic in south-east Asia and, in the last few years, has become a severe pest to fruit growers in North and South America and Europe^3^.

In contrast to most of the other fruit flies in the invaded regions, females of SWD have a serrated ovipositor^4^, enabling them to lay eggs in healthy and undamaged fruits^5^. *D. suzukii* can reproduce on a broad range of wild and cultivated soft-skinned fruit crops and can have an extremely high reproduction rate^6^. Therefore, enormous populations can be build up quickly and infest fruit crops, where they cause massive economic damage^7^.

The control of this *Drosophila* is still firmly based on the use of insecticides due to the lack of effective alternatives. Here, biological options, such as predators, parasitoids, nematodes, bacteria, fungi and viruses, could be a possible part of an Integrated Pest Management strategy (IPM)^8,9^ and interest in them has been growing.

Parasitoids are, in particular, a promising option because, in natural systems, parasitisation rates of *Drosophila* can reach up to 50%^10^. Parasitoid wasps from the native range of SWD show high efficacy and specialisation on the pest flies^11–15^. Whereas some of these species were under consideration to be introduced to North America and Europe, first specimens were already discovered in North America^16^ and Europe^17^. However, the larval parasitoids native on these continents are not able to successfully reproduce on *D. suzukii*^18,19^. The most promising native parasitoids in Europe and North America that can successfully reproduce on *D. suzukii,* are the pupal parasitoids *Pachycrepoideus vindemmiae* Rondani (Hymenoptera: Pteromalidae) and *Trichopria drosophilae* Perkins (Hymenoptera: Diapriidae)^19^. Ideally, these wasps should be implemented in an IPM approach.

For an augmentative release, knowledge of the species’ quality parameters, such as host identification, specificity to the host, the ratio of parasitism, the ratio of emergence (e.g., ≥ 90% for trichogrammatids) and the ratio of females (≥ 50%) is essential^20^. Furthermore, the release of parasitic wasps should happen as early as possible in the growing season, when the population size of *Drosophila* is still small^21,22^.

*Trichopria drosophilae* is the most promising candidate for augmentative biocontrol of SWD and is already available on the market^23,24^. This wasp species has a high foraging efficiency on *D. suzukii* pupae and a high load of mature eggs^19,23–29^. Furthermore, *T. drosophilae* can parasitise at lower temperatures (8–25 °C) than the species *P. vindemmiae*^30–32^. This early parasitisation is an advantage when implementing an IPM program as it can parasitise the first generations of the pest early in the year. *T. drosophilae* can parasitise a broad host range of many Drosophilidae species^33^, including the widespread fly *D. melanogaster* Meigen (Diptera: Drosophilidae).

This fly is not a pest of healthy fruits and consequently not a target of the IPM approach, similar to most other Drosophilidae species. For an efficient IPM, it is therefore essential to study the host specificity and the host identification mechanism of *T. drosophilae*.

A preference of *T. drosophilae* for *D. suzukii* over *D. melanogaster* has been observed in several studies^34–36^, although one study has found no differences in parasitisation between the two species^23^. However, *D. suzukii* pupae are larger than pupae of *D. melanogaster*, at least under optimal food supply. Therefore, it is uncertain if this observed preference of *T. drosophilae* for *D. suzukii* is due to the larger host pupal size or to the host itself. Furthermore, the pupal size is an unsteady factor for host selection as varying food supply under natural conditions can lead to a high variation in pupal size.

Until today studies have used indirect measures for the oviposition preference of wasps on SWD, such as the number of emerged parasitoid wasps or the degree of infestation (DI)^34–36^. These measurements have uncertainties, primarily due to the immune response of the fly species, which can kill the wasp eggs oviposited into the larvae or pupae^37^. Therefore, the number of emerged wasps is usually smaller than the number of deposited eggs. To accurately study the host and oviposition preference, the oviposition events must be observed directly.

In this work, we studied the host preference of *T. drosophilae* for *D. suzukii* taking the size of the pupae and the immune response of the fly into account. To correct for pupal size, we measured the size of each pupa, and by direct observation of each oviposition, we determined the real oviposition preference.

This way, we provide evidence for the host-choice of *T. drosophilae* and contribute essential new knowledge about its behaviour during oviposition as well as about offspring sex-ratios of this promising biological control agent. With these new results, effective use of *T. drosophilae* wasps becomes more feasible, and the negative impacts of the invasive *D. suzukii* flies on crops could be decreased.

## Material and methods

### Insects

The fly species *D. suzukii and D.* *melanogaster* were used for oviposition preference tests of *T. drosophilae*. The strain of *D. suzukii* was caught in the state of Hesse, Germany, in 2016 and was refreshed in 2017. The strain of *D. melanogaster* is an established lab strain for multiple generations. *D. melanogaster* was reared on an artificial Drosophila diet (ingredients: 1 l water, 50 g cornmeal, 50 g wheat germ, 50 g sugar, 40 g baker’s yeast, 8 g agar, 5 ml propionic acid, 20 ml methylparaben (10%)) in Drosophila vials.

Adult *D. suzukii* flies were kept in a BugDorm cage (MegaView Science Co., Taichung, Taiwan), where 10% of sugar water was provided ad libitum. The flies were allowed to lay eggs in Drosophila vials with the same artificial diet as *D. melanogaster*. Then the vials were removed from the cages. The fly development took place in these vials until the flies emerged. After some days, they were released into the cage. Variation in pupae volume was created by rearing both fly species with a higher and lower density of larvae per amount of diet.

The parasitoid wasp *T. drosophilae* was provided by the company “Bioplanet” in Cesena, Italy. In the lab, two different populations were reared for more than two years (approx. > 40 generations) in Drosophila vials on pupae of either *D. melanogaster* or *D. suzukii*, henceforth referred to as *T. drosophilae* < melanogaster > and *T. drosophilae* < suzukii > , respectively. After the wasps’ emergence, they were fed with a 10% honey-water solution. The parasitoid females used for the experiments were 4–6 days old and were held together with males. All insects were kept in a climate- and light-controlled chamber at 24 °C and 70% to 80% RH with a 16:8 h day to night rhythm.

### Host preference experiments

In a choice experiment, we wanted to test whether females of *T. drosophilae* prefer to oviposit in pupae of *D. suzukii* over *D. melanogaster*. For this, 15 pupae of each *D. suzukii* and *D. melanogaster* were arranged alternately (*D. suzukii* pupae next to *D. melanogaster* pupae and so on) on a disk of moist filter paper which was placed in a Petri dish (9.5 cm diameter). We increased the variation in the size of the pupae by rearing both fly species (*D. melanogaster* and *D. suzukii*) with a higher or lower amount of food per larvae. To accurately measure the size of the pupae, the Petri dish was photographed (Canon Eos M100) next to a precision ruler for scale. The length and width of each pupa were measured with the software ImageJ^38^ from the photos, and the volume was calculated using the formula^39^:

1 $$V=\frac{4}{3}\pi \cdot \frac{l}{2}\cdot {\left(\frac{w}{2}\right)}^{2}$$

where $V$ is the volume, $l$ the length and $w$ the width of the pupae.

One female of *T. drosophilae* was added to each Petri dish, and the wasp oviposition was recorded using a digital video recorder (Lupustec LE 800 4 K, LupusElectronics GmbH, Landau, Germany) for six hours. To reduce possible self-superparasitisation, we used a shorter exposition time of the wasp to SWD pupae than in previously conducted studies^23,34–36^. The added female of *T. drosophilae* was either reared on *D. melanogaster* (*T. drosophilae* < melanogaster >) or *D. suzukii* (*T. drosophilae* < suzukii >). The oviposition events (host species and duration) were analysed using the event logging software BORIS^40^. An oviposition event was logged when the wasp pierced a pupa and did not move during that behaviour for a minimum of 30 s.

Eight Petri dishes were prepared for each wasp treatment. As a control, no wasps were added to eight Petri dishes with fly pupae. After the potential oviposition, the pupae were transferred individually to 96-well plates and the species (*D. melanogaster*, *D. suzukii*, or *T. drosophilae*) and the sex of the emerged insects were recorded. When only male wasps hatched from a repetition, it was assumed that the female wasp was unmated, and the repetition was excluded from the analysis. All experiments were conducted in the same climate-controlled chamber, under the same conditions as for the insects rearing (see “Insects”).

### Sex ratio of emerged parasitoids

Previous studies observed a female-biased offspring sex ratio for wasps emerging from *D. suzukii* compared to *D. melanogaster*^34–36^. However, the size of the individual pupae was not measured in those studies. To confirm that the sex ratio of *T. drosophilae* is pupal-size dependent, we recorded in both experiments the sex of the emerged parasitoids. The measured size of each pupa was then used to determine this dependency.

### Statistical analysis

The effect of the *Drosophila* species *D. suzukii* and *D. melanogaster* and of the pupal size on the number of parasitised pupae and the number of successful parasitisations by *T. drosophilae* females was analysed using a binomial generalised linear mixed model (GLMMs) in the R package lme4^41^. The model was used for the effect of pupal size on the sex of emerged wasps. As the interaction between wasp type and host species in the GLMM was not significant, it was excluded from further analyses. Separate GLMMs for each wasp type were performed. Female wasps without observed parasitisation or with only male offspring were excluded from testing. The parasitised pupae and number of successful parasitisation events for each wasp treatment and host species were compared using the Wilcoxon rank-sum test. Data were analysed in R 3.6.1^42^.

## Results

### Observed oviposition preference

**Figure 1:**
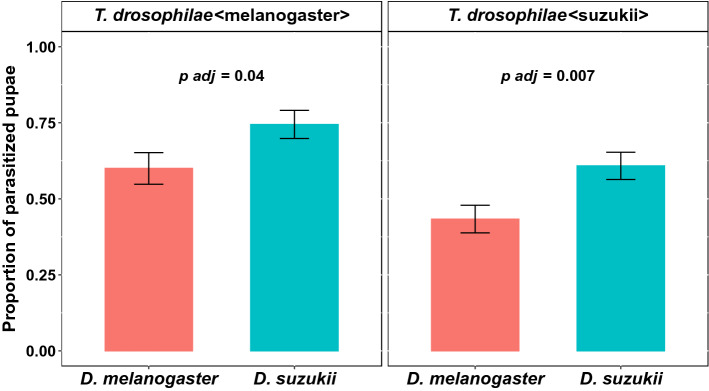
Proportion of parasitised pupae of *D. suzukii* (blue) and *D. melanogaster* (red) by the wasp *T. drosophilae*. The wasp was reared on either *D. melanogaster* pupae (left side) or *D. suzukii* pupae (right side) (Wilcoxon rank-sum test).

When the *T. drosophilae* females from the two populations (reared either on *D. melanogaster* or on *D. suzukii*) had the choice between *D. suzukii* and *D. melanogaster* as a host, significantly more pupae of *D. suzukii* were parasitised (*T. drosophilae* < melanogaster > W = 3465, *p* *adj* = 0.04; *T. drosophilae* < suzukii > W = 5940, *p* *adj* = 0.007, Fig. 1). Independent of the host species, *T. drosophilae* < melanogaster > parasitised significantly more pupae than *T. drosophilae* < suzukii > (Wilcoxon rank-sum test; W = 24,870, *p* = 0.002).

**Figure 2:**
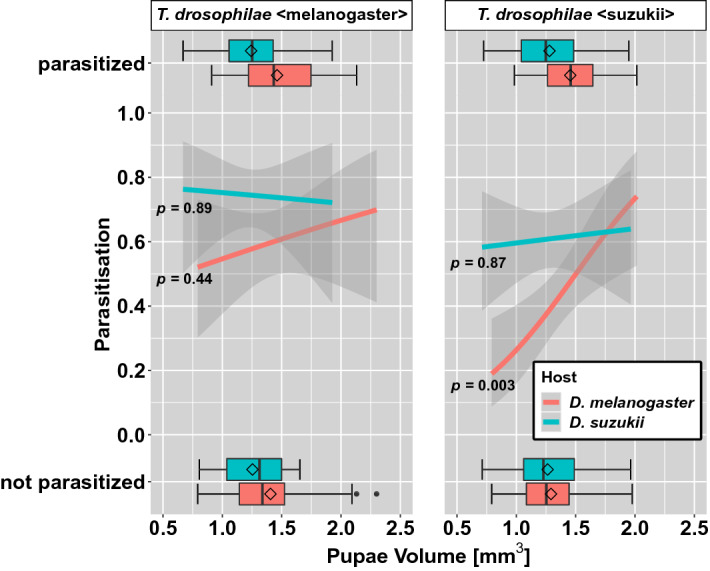
Proportions with a 95% confidence interval of parasitised *D. suzukii* (blue) and *D. melanogaster* (red) pupae in relation to pupae volume. The wasp *T. drosophilae* was reared either on *D. melanogaster* (left side) or *D. suzukii* pupae (right side). The variation of the volume of parasitised and not parasitised pupae volume is given in the box plots on top and bottom (for GLMMs see Table S1).

The preference of *T. drosophilae* < melanogaster > was not influenced by the pupal size of *D. suzukii* (*p* = 0.89) and *D. melanogaster* (*p* = 0.44) (Fig. 2). The *T. drosophilae* < suzukii > preference was also not influenced by the pupal size of *D. suzukii* (*p* = 0.89). It was, however, influenced significantly by the size of the *D. melanogaster* pupae (*p* = 0.003; Fig. 2, Table S1).

### Parasitisation success

**Figure 3:**
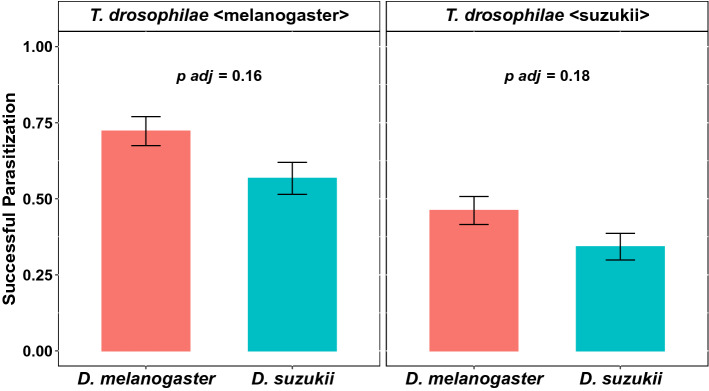
Mean (± SEM) proportion of successful parasitisation of pupae of *D. suzukii* (blue) and *D. melanogaster* (red) by the wasp *T. drosophilae*. The wasp was reared on *D. melanogaster* (left side) pupae and *D. suzukii* pupae (right side). No significant differences were observed between the successful parasitisation of pupae of *D. melanogaster* and *D. suzukii* with females of *T. drosophilae* reared on *D. melanogaster* or *D. suzukii* (Wilcoxon rank-sum test).

The number of emerged wasps out of previously parasitised pupae is given by the parasitisation success. *T. drosophilae* tend to have a higher parasitisation success in pupae of *D. melanogaster* compared to those of *D.* *suzukii*, although the difference is not significant (W = 2089, *p adj* = 0.16 and W = 2124, *p adj* = 0.18, Fig. 3). Independent of the pupae species, the wasp strain reared previously on *D. melanogaster* (*T. drosophilae* < *melanogaster* >) had a significantly higher parasitisation success than *T. drosophilae* reared on *D. suzukii* (*T. drosophilae* < suzukii > ; Wilcoxon rank-sum test; W = 9410.5, *p* < 0.001).

**Figure 4:**
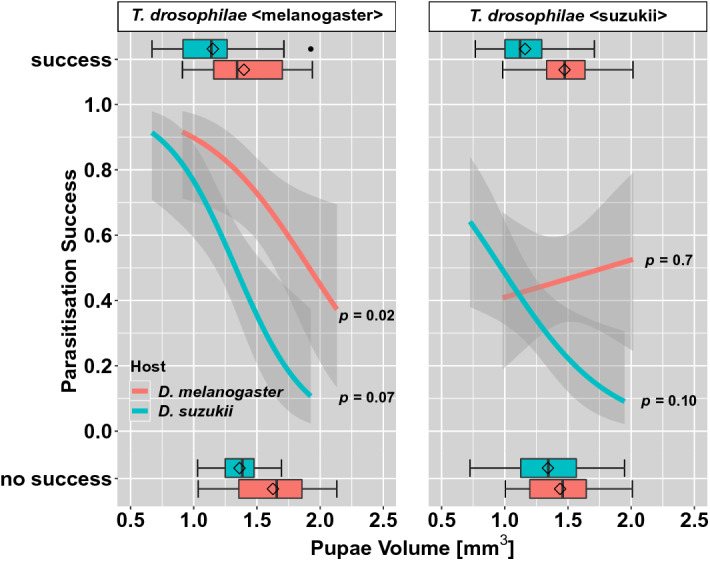
Proportions with a 95% confidence interval of parasitisation success on *D. suzukii* (blue) and *D. melanogaster* (red) pupae in relation to pupal volume. The wasp *T. drosophilae* was reared on *D. melanogaster* pupae (left side) and *D. suzukii* pupae (right side). The variation of success and no success of parasitisation to the volume of parasitised pupae is given in the box plots on top and bottom (for GLMMs see Table S2).

The parasitisation success of *T. drosophilae* < melanogaster > was negatively influenced by the pupal volume of *D. melanogaster* pupae (Fig. 4, Table S2). This means that the probability of successful development of a wasp decreases with increasing pupal size of flies. Also, with an increasing pupal size of *D.* *suzukii* pupae, a visible tendency was observed for a decrease in the successful parasitisation of *T. drosophilae* < melanogaster > . The parasitisation success of *T. drosophilae* < suzukii > was not influenced by the pupal size of *D. melanogaster* pupae. However, in *D. suzukii* pupae, a slight tendency is observed that the successful parasitisation decreases with increasing pupal volume.

### Emergence of *Drosophila* from parasitised pupae

**Figure 5:**
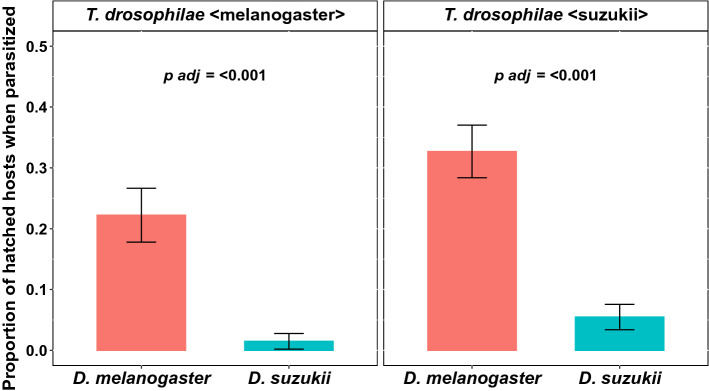
Mean (± SEM) Proportions of emerged hosts from parasitised hosts. The hosts were pupae of *D. suzukii* (blue) and *D. melanogaster* (red), parasitised from the wasp *T. drosophilae*. The wasp was reared on *D. melanogaster* (left side) pupae and *D. suzukii* (right side) pupae. In both wasp populations, *D. melanogaster* had a significantly higher proportion of emerged flies than *D. suzukii* (Wilcoxon rank-sum test).

To evaluate whether the high parasitisation success of the parasitoid, as described in “Parasitisation success”, also means a lower probability that flies emerged out of the parasitised pupae, we calculated the proportion of emerged flies out of the parasitised pupae. On average, 20–30% flies emerged from the *D. melanogaster* pupae parasitised from the two different reared wasp strains. For the parasitised *D. suzukii* pupae, in both wasp strains, significantly fewer flies emerged (*T. drosophilae* < *melanogaster* > *:* W = 2184, *p adj* =  < 0.001; *T. drosophilae* < suzukii > : W = 2414, *p adj* =  < 0.001, on average 1–5% (Fig. 5).

### Sex of emerged wasps depending on host pupal volume

**Figure 6:**
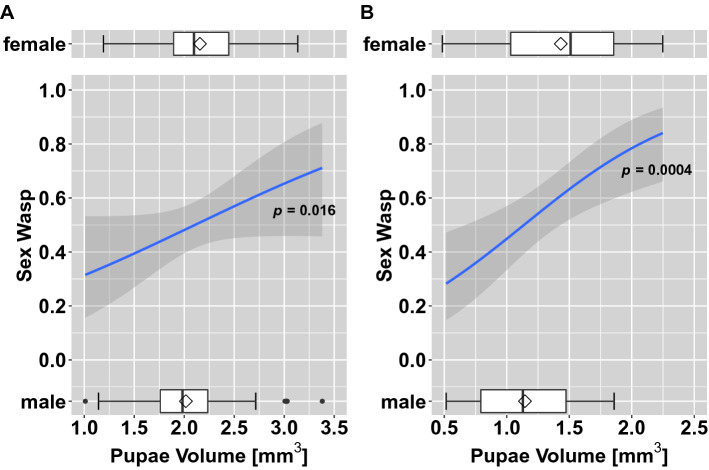
Sex of emerged *T. drosophilae* from a no choice test situation depending on the host pupal volume of A: *D. suzukii* and B: *D. melanogaster*. Boxplots give the variation in size for the pupae from which male and female wasps emerged. The curve is an estimated proportion of the sex as a function of pupal volume with a 95% confidence interval (binomial GLMM, Table S3).

**Figure 7:**
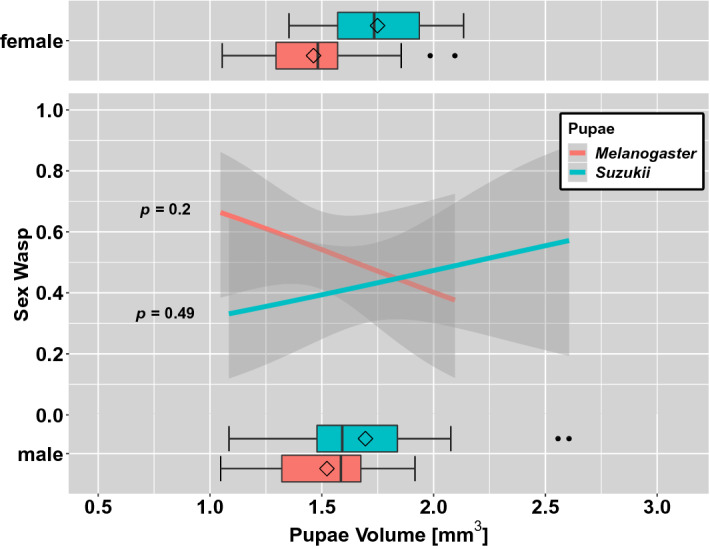
Sex of emerged *T. drosophilae* from a choice test situation depending on the host pupal volume of *D melanogaster* (red) and *D. suzukii* (blue). Boxplots give the variation in size for the pupae from which male and female wasps emerged. Curves are estimated proportions of the sex as a function of pupal volume with a 95% confidence interval (binomial GLMM, Table S3).

To see if the sex ratio of emerged *T. drosophilae* can be potentially modified, we plotted the sex ratio of the wasps to the volume of the pupae, out of which the wasps emerged. The pupae volume of *D. suzukii* and *D. melanogaster* in a no-choice situation had a significant increasing effect on the female-biased sex ratio (Fig. 6). However, when the wasps had the choice between the pupae of both *Drosophila* species (*D. melanogaster* and *D.* *suzukii*), the pupal size did not affect the sex ratio (Fig. 7). Furthermore, the sex ratios of the emerged wasps in the two *Drosophila* species were not significantly different, under the choice test set up (post hoc Tukey test: *p* = *0.178*).

## Discussion

Our results show that the parasitoid wasp *T.* *drosophilae* has an oviposition preference for pupae of the invasive pest *D. suzukii* over those of the widespread fly *D. melanogaster*. The preference for the invasive pest was regardless of the host species on which the wasps were reared. Furthermore, we can exclude the pupal volume as the reason for that species preference because the pupal size did not affect the oviposition preference, except for *D. melanogaster* pupae parasitised by *T. drosophilae* reared on *D. suzukii*. Even when the pupae of the two *Drosophila* species were adjusted to be similar in size, there was still a significant preference for the *D. suzukii* pupae. In total, we can conclude that the choice of *T. drosophilae* wasps for *D. suzukii* is a real preference for the species and not a preference for larger pupae as concluded in some studies^34,36^.

A preference of *T. drosophilae* for *D. suzukii* was reported in previous studies^34–36^. However, those studies did not measure the pupal size, and the preference was based on the number of emerged wasps and flies, not on direct observation of oviposition. This difference is important because our data show that direct observations are more accurate than the traditionally used measures: the degree of infestation (DI), which measures the proportion of successfully parasitised hosts, and the success of parasitism (SP), which measures the proportion of emerged wasps out of parasitised pupae. The DI was always higher than the here observed oviposition, and the SP was always lower than the here observed parasitisation success (Figure S1, Figure S2). DI and SP are consequently less adequate when evaluating wasp parasitisation success. One of the reasons is that defining the preference of a parasitoid by the number of emerged parasitoids does not take into account that a wasp larva has different success rates in different *Drosophila* hosts. Direct observation of the parasitoid’s preference using video recordings should thus be the preferred method for an accurate analysis of parasitisation of fly pupae.

One possibility of how parasitoids wasps can distinguish between pupae of different host species is that they could use species-specific chemical cues of the pupae^43^. Romani, et al.^44^ observed for *T. drosophilae* that the host's chemical cues of the anterior spiracles of *D.* *melanogaster* are probably the most important cue for host recognition. In *D. suzukii* pupae, these anterior spiracles have seven to eight radially arranged branches^6^ and are thus more structured than they are in *D.* *melanogaster*. The anterior respiratory spiracles are especially crucial because the anterior part of the pupae of *D. suzukii* is orientated outside of fruit or soil, and the soil is the most common pupation location of SWD^45^. Therefore, this is the only part of the pupae the wasp antenna has physical contact to during the searching process and, consequently, it may be crucial for the pupae recognition and perhaps pupae species recognition.

Furthermore, we noticed during observation of the parasitisation that *T. drosophila* wasps seem to make their decision to parasitise predominantly after drumming with their antennae over respiratory spiracles of the pupae. This *T. drosophilae* behaviour was also observed by Romani, et al.^44^ and is in accordance with other studies stating that, during direct contact of the parasitoid with the host, the host’s form and texture are essential for host selection and acceptance^46,47^. So, we can assume that the preference for *D. suzukii* is probably due to a combination of chemical and physical cues of the pupa’s anterior spiracles, which mediates the parasitoid’s recognition of a host.

The lower parasitisation success rates of the *T. drosophilae* wasp in *D. suzukii* compared to those in *D. melanogaster* could be due to a different immune resistance of the two fly species. Kacsoh and Schlenke^18^ and Poyet, et al.^48^ found a higher haemocyte load and lower encapsulation rates in *D. suzukii* larvae than in *D. melanogaster* larvae when they were parasitised with larval parasitoids. Furthermore, at least in *D. melanogaster,* the immune system of the pupae is different from the larval immune system, for example, the haemocytes undergo morphological changes in the pupal stage^49^. The immune system of *D.* *suzukii* pupae has not yet been studied, but we observed these morphological changes of the haemocytes also in *D. suzukii* pupae (unpublished results).

The lower success rate of *T. drosophilae* in pupae of *D. suzukii* does not benefit the host. It was exceptional for an adult fly to emerge out of a parasitised *D. suzukii* pupa. Such high mortality of flies during their development was not observed for *D. melanogaster.* This lack of survival advantage for *D. suzukii* was also observed by Kacsoh and Schlenke^18^ and Iacovone, et al.^50^. The reason that nearly no parasitised pest fly emerged could be a hyperactive immune system in *D. suzukii*, as a hyper-activation of the JAK/STAT signalling pathway was observed to trigger self-encapsulation in *D. melanogaster* larvae^51^.

However, self-encapsulation was not observed until now in pupae of *Drosophila* and is unlikely because the key haemocytes for encapsulation, the lamellocytes, are no longer present in the pupae and cannot be induced by injury^49^. Although the larval immune system of *D. suzukii* is known to resist parasitoids strongly, this effect has not yet been studied for its pupae. Further research is needed to determine whether, in general, the pupae of *D. suzukii* also have in comparison to other *Drosophila* a stronger resistance (low parasitisation success) against pupal parasitoids. It remains unclear whether the survival disadvantage of the pest fly is due to a hyperactive immune system or possibly due to the venom of the pupal parasitoid injected during parasitisation, which affects the *D. suzukii* pupae.

In parasitisation tests of *T. drosophilae* on pupae of different volume of either *D. melanogaster* or *D. suzukii* (no-choice tests), the sex of the emerged wasp depends on the pupal size: Out of larger hosts, female parasitoids predominately emerged, whereas male wasps predominantly emerged out of smaller hosts. So, the probability that a diploid (fertilised) egg was oviposited by a parasitoid female increased with increasing host pupal volume (Fig. 6). In several parasitoid systems, it was observed that the sex ratio shifts in larger hosts to be more female-biased^52–54^. The larger hosts give increased fitness for both sexes of the parasitoid; however, this increase is greater for female wasps than for male wasps^52^. Consequently, an ovipositing female should lay female offspring in larger hosts, which is what we observed for *T. drosophilae*.

However, in choice tests where wasps could decide between differently sized pupae of the two host species, we found no effect of the host size on the sex of the parasitoids offspring. This could be due to the decision to oviposit in different species being dominant over the sex ratio adjustment of a female *T. drosophilae*. Therefore, the effect of host size on sex ratio is missing in the species choice test. Boycheva Woltering, et al.^35^ also found a higher female-biased sex ratio for *T. drosophilae* emerging from *D. suzukii* pupae than from those of *D. melanogaster* or *D. immigrans* under choice situations. In a no-choice situation, the sex ratios were similar for all three hosts. However, in those tests, they did not adjust the host size or measured the variance in host size. Therefore, in their study, the effect of host size on the sex ratio of the parasitoid offspring remains unclear.

For a mass-rearing, a high female-biased sex ratio is beneficial, especially for the last wasp generation, which will be released into the field. An adjustment to a higher female-biased sex ratio appears to be achievable by rearing the wasps on hosts with larger pupal sizes. Furthermore, the species for mass rearing can be *D. melanogaster* which is more accessible and the host species does not appear to negatively influence the parasitoid’s preference for *D. suzukii*.

## Conclusion

In the last years, the pest species *D. suzukii* causes massive agricultural losses worldwide. An effective control agent for *D. suzukii* in an IPM approach could be the parasitoid wasp *T. drosophilae*.

Here, a preference of *T. drosophilae* for the pest is essential for the success of a parasitoid release under field conditions. We could show that *T. drosophilae* has a significant preference for *D. suzukii* and that this preference is independent of the pupae size and the fly species on which the wasps were reared. Also, the probability of a successful parasitisation of *T. drosophilae* is not affected by the previous hosts. We, therefore, conclude that, for mass rearing of the wasps, there is no benefit from using *D. suzukii* as a host. Instead, *D. melanogaster*, which is easier to handle and to rear, can be used to mass-rear the wasps.

The efficiency of a parasitoid wasp as a biocontrol agent also depends on the ratio of female wasps. Here we show that large-sized *D. melanogaster* pupae can be used to increase the proportion of female *T. drosophilae*, reared either on *D. suzukii* or *D. melanogaster*.

Overall, our study showed that *T. drosophilae* is a generalist parasitoid with a preference for *D. suzukii* over the very common fly *D. melanogaster*. This preference makes this parasitoid an even more promising candidate as a biocontrol agent in an IPM for *D. suzukii*.

## Supplementary Information

Supplementary Information 1.

Supplementary Information 2.
