# Supplementary material for: The preference of Trichopria drosophilae for pupae of Drosophila suzukii is independent of host size
Source: Sci Rep. 2021 Jan 13;11:995. doi: 10.1038/s41598-020-80355-5 (PMC7806991; doi:10.1038/s41598-020-80355-5)
Supplement: Supplementary file 1 — Supplementary Information 1. [file 41598_2020_80355_MOESM1_ESM.pdf]

Supplementary Material

The Preference of *Trichopria drosophilae* for pupae of *Drosophila suzukii* is independent of the host size

Benedikt J. M. Häußling[1*], Judith Lienenlüke[2], Johannes Stökl[1]

[1]Department of Evolutionary Animal Ecology, Bayreuth University, Bayreuth, Germany

[2]Institute for Insect Biotechnology, Justus-Liebig University Giessen, Germany

*Corresponding author: Benedikt.Haeussling@uni-bayreuth.de

ORCID:

Benedikt J.M. Häußling: 0000-0003-1737-6824

Johannes Stökl: 0000-0002-6471-434X

Supplementary

Table S1: Observed oviposition preference – Generalised linear mixed effect model (family = *binomial*, link = logit, "Petri dish" as a random factor) output quantifying the effect of pupae size on the parasitisation of *T. drosophilae*, reared on *D. melanogaster* (*T. d.<mel>*) or on *D. suzukii* (*T. d <suz>),* in the pupae of *D. melanogaster* (*D. mel*) or *D. suzukii* (*D. suz*).

**Subset**	**Factor**	χ^2	*df*	***p*-value**
T. d. <mel> D. mel	Pupae size	0.5908	1	0.442
T. d. <mel> D. suz	Pupae size	0.0205	1	0.886
T. d. <suz> D. mel	Pupae size	8.7012	1	0.003
T. d. <suz> D. suz	Pupae size	0.0285	1	0.866

Table S2: Parasitisation success – Generalised linear mixed effect model (family = binomial, link = logit, "Petri dish" as a random factor) output quantifying the effect of pupal size on the success of parasitisation of the wasp *T. drosophilae*, reared on *D. melanogaster* (*T. d.<mel>*) or on *D. suzukii* (*T. d <suz>),* in the pupae of *D. melanogaster* (*D. mel*) or *D. suzukii* (*D. suz*).

**Subset**	**Factor**	χ^2	*df*	***p*-value**
T. d. <mel> D. mel	Pupae size	5.0907	1	0.024
T. d. <mel> D. suz	Pupae size	3.4036	1	0.065
T. d. <suz> D. mel	Pupae size	0.1538	1	0.695
T. d. <suz> D. suz	Pupae size	2.697	1	0.101

Table S3: Sex ratio to the pupal volume of pupae of – Generalised linear mixed effect model (family = binomial, link = logit, "Petri dish" as a random factor) output quantifying the effect of pupae size on the sex ratio of emerged *T. drosophilae*

**Sample / subset**	**Factor**	χ^2	*df*	***p*-value**
**No choice test**				
D. suzukii	Pupal size	5.8557	1	0.155
D. melanogaster	Pupal size	12.557	1	0.0004
D. virilis	Pupal size	3.5909	1	0.058
**Choice test**				
D. suzukii	Pupal size	0.476	1	0.49
D. melanogaster	Pupal size	1.6829	1	0.195

<u>Comparison Degree of Infestation with Parasitation Video</u>

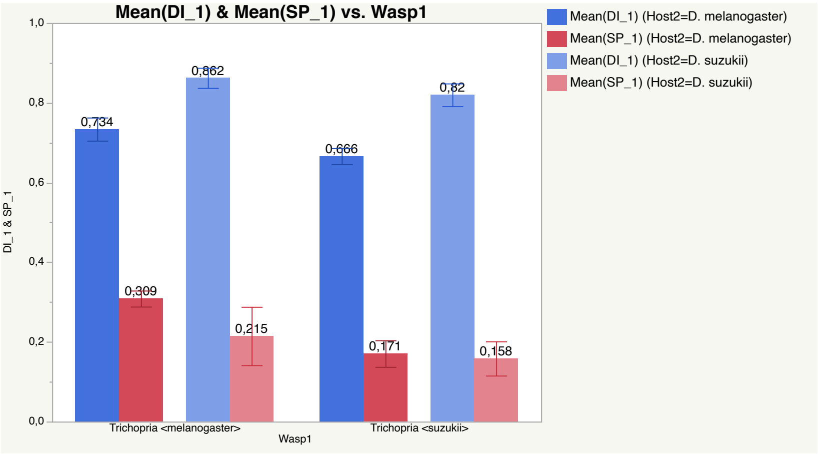

Figure S1: Degree of Infestation (DI) (blue) and success of parasitism (SP) (red) of *Trichopria drosophila* on the host *Drosophila melanogaster* and *Drosophila suzukii*.

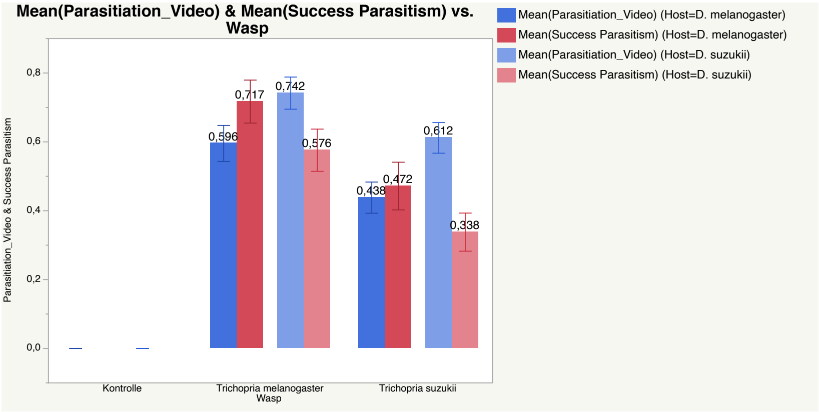

Figure S2: The mean observed parasitised pupae (blue) and the success of the parasitised wasp (*Trichopria drosophila*) to hatch from the host *Drosophila melanogaster* and *Drosophila suzukii.*
