# Supplementary material for: The preference of Trichopria drosophilae for pupae of Drosophila suzukii is independent of host size
Source: Sci Rep. 2021 Jan 13;11:995. doi: 10.1038/s41598-020-80355-5 (PMC7806991; doi:10.1038/s41598-020-80355-5)
Supplement: Supplementary file 2 — Supplementary Information 2. [file 41598_2020_80355_MOESM2_ESM.pdf]

R Notebook

This code is also avaible at github.com/Benehaeus/Suzukiipref

Basic system setup

Clean up and install/load packages

If not yet installed, pacman will install and load necesery packages.

```
rm(list = ls())
if(!require('pacman'))install.packages('pacman')
```

```
## Loading required package: pacman
```

```
pacman::p_load(readxl,
               lme4,
               car,
               ggplot2,
               cowplot,
               stringr,
               MuMIn,
               ggpubr,
               multcomp,
               plyr,
               reshape2)
```

ETL - extract, transform, load

Extract: read from excel file

```
# place all data from local directory
Daten <-
  read_excel(
    "Data_Pupae_size_video.xlsx",
    sheet = "Tabelle1",
    na = "NA"
  )
```

Transform:

```
Daten$Repetition <- as.factor(Daten$Repetition)
Daten$Puppae <- as.factor(Daten$Puppae)
Daten1 <-
  subset(Daten,
         Wasp == 'T. drosophilae <suzukii>' |
           Wasp == 'T. drosophilae <melanogaster>')
#exclude Repetitons which had faults
Daten2 <- Daten1[Daten1$Repetition %in% c(11:25, 27:28),]
Daten2$Parasitiation_Video_factor <-
  str_replace_all(as.factor(Daten2$Parasitiation_Video), "0", "not parasitized")
Daten2$Parasitiation_Video_factor <-
```

```
  str_replace_all(as.factor(Daten2$Parasitiation_Video_factor),
                  "1",
                  "parasitized")
Daten2$Parasitiation_Video_factor <-
  as.factor(Daten2$Parasitiation_Video_factor)

is.factor(Daten2$Parasitiation_Video_factor)
Daten2$Parasitation_succes <-
  as.numeric(as.character(Daten2$Parasitation_succes))
is.numeric(Daten2$Parasitation_succes)

Daten2$Fly_success_parasitization <-
  as.numeric(as.character(Daten2$Fly_success_parasitization))
is.numeric(Daten2$Fly_success_parasitization)

Daten2$Parasitiation_success_factor <-
  str_replace_all(as.factor(Daten2$Parasitation_succes), "0", "no success")
Daten2$Parasitiation_success_factor <-
  str_replace_all(as.factor(Daten2$Parasitiation_success_factor),
                  "1",
                  "success")
Daten2$Parasitiation_success_factor <-
  as.factor(Daten2$Parasitiation_success_factor)
is.factor(Daten2$Parasitiation_success_factor)

subsetTriSuz <- subset(Daten2, Wasp == 'T. drosophilae <suzukii>')
subsetTriSuz_Mel <- subset(subsetTriSuz, Host == 'D. melanogaster')
subsetTriSuz_Suz <- subset(subsetTriSuz, Host == 'D. suzukii')

subsetTriMel <-
  subset(Daten2, Wasp == 'T. drosophilae <melanogaster>')
subsetTriMel_Mel <- subset(subsetTriMel, Host == 'D. melanogaster')
subsetTriMel_Suz <- subset(subsetTriMel, Host == 'D. suzukii')

subsetMel <- subset(Daten2, Host == 'D. melanogaster')
subsetMelTriMel <-
  subset(subsetMel, Wasp == 'T. drosophilae <melanogaster>')
subsetMelTriSuz <-
  subset(subsetMel, Wasp == 'T. drosophilae <suzukii>')
subsetSuz <- subset(Daten2, Host == 'D. suzukii')
subsetSuzTriSuz <-
  subset(subsetSuz, Wasp == 'T. drosophilae <suzukii>')
subsetSuzTriMel <-
  subset(subsetSuz, Wasp == 'T. drosophilae <melanogaster>')
```

Functions and presets

design presets

Some plots share their design, which is set up here

```
theme_wasp <- function() {
  theme_gray() +
```

```
    theme(
      panel.background = element_rect(color = "lightgray", fill = "lightgray"),
      plot.title = element_text(
        color = "black",
        face = "bold",
        size = 18,
        hjust = 0.5
      ),
      axis.title = element_text(
        color = "black",
        face = "bold",
        size = 18
      ),
      axis.text = element_text(color = "black", size = 18, face = "bold"),
    )
}
```

Data analysis

GLM Test Parasitaziation Video

```
#sink('GLM-Geschlecht_Wespe_virilis-output.txt')
m1 <-
  glmer(
    Parasitiation_Video ~  Host * Volume * Wasp + (1 |
                                                    Repetition),
    data = Daten2,
    family = binomial
  )
r.squaredGLMM(m1)
summary(m1)
Anova(m1)
summary(glht(m1, mcp(Host = "Tukey", Wasp = "Tukey")))
#sink()
```

```
#sink('GLM-Geschlecht_Wespe_virilis-output.txt')
m3 <-
  glmer(
    Parasitiation_Video ~ Volume + Wasp + (1 |
                                            Repetition),
    data = subsetMel,
    family = binomial
  )
```

```
## boundary (singular) fit: see ?isSingular
```

```
r.squaredGLMM(m3)
summary(m3)
Anova(m3)
summary(glht(m3, mcp(Wasp = "Tukey")))
#sink()
#sink('GLM-Geschlecht_Wespe_virilis-output.txt')
m6 <-
  glmer(
```

```
    Parasitiation_Video ~ Volume + Wasp + (1 |
                                          Repetition),
    data = subsetSuz,
    family = binomial
  )
r.squaredGLMM(m6)
summary(m6)
Anova(m6)
summary(glht(m6, mcp(Wasp = "Tukey")))
#sink()
```

GlM Melanogaster Pupae Parasitation of T. drosophilae

```
#sink('GLM-Geschlecht_Wespe_virilis-output.txt')
m4 <-
  glmer(Parasitiation_Video ~ Volume  + (1 |
                                          Repetition),
        data = subsetMelTriSuz,
        family = binomial)
```

```
## boundary (singular) fit: see ?isSingular
```

```
r.squaredGLMM(m4)
summary(m4)
Anova(m4)
#sink()

m5 <-
  glmer(Parasitiation_Video ~ Volume  + (1 |
                                          Repetition),
        data = subsetMelTriMel ,
        family = binomial)
```

```
## boundary (singular) fit: see ?isSingular
```

```
r.squaredGLMM(m5)
```

```
## boundary (singular) fit: see ?isSingular
```

```
summary(m5)
Anova(m5)
#sink()
```

```
#sink('GLM-Geschlecht_Wespe_virilis-output.txt')
m7 <-
  glmer(
    Parasitiation_Video ~ Volume  + (1 |
                                    Repetition),
    data = subsetSuzTriSuz,
    family = binomial
  )
    r.squaredGLMM(m7)
    summary(m7)
    Anova(m7)
    #sink()
```

```r
m8 <-
  glmer(
      Parasitiation_Video ~ Volume  + (1 |
                                         Repetition),
        data = subsetSuzTriMel ,
        family = binomial
      )
    r.squaredGLMM(m8)
    summary(m8)
    Anova(m8
    )
#sink()
```

```r
#sink('GLM-Geschlecht_Wespe_virilis-output.txt')
m2 <-
  glmer(
    Time_of_parasitation_sec ~ Volume + Host + Wasp * +(1 |
                                                        Repetition),
    data = Daten2,
    family = gaussian
  )
```

```
## boundary (singular) fit: see ?isSingular
```

```r
r.squaredGLMM(m2)
summary(m2)
Anova(m2)
#sink()
```

GLM Preference of different reared Trichopria

```r
#sink('GLM-Geschlecht_Wespe_virilis-output.txt')
m9 <-
  glmer(
    Parasitiation_Video ~  Host * Volume + (1 |
                                            Repetition),
    data = subsetTriSuz,
    family = binomial
  )
r.squaredGLMM(m9)
summary(m9)
Anova(m9)
summary(glht(m9, mcp(Host = "Tukey")))
#sink()

m10 <-
  glmer(
    Parasitiation_Video ~  Host * Volume + (1 |
                                            Repetition),
    data = subsetTriMel ,
    family = binomial
  )
```

```
## boundary (singular) fit: see ?isSingular
```

```
r.squaredGLMM(m10)
```

```
## boundary (singular) fit: see ?isSingular
```

```
summary(m10)
Anova(m10)
summary(glht(m10, mcp(Host = "Tukey")))
#sink()
```

Significanc test Degree of Infastation

```
compare_means(Parasitiation_Video ~ Wasp, data = Daten2, method = "wilcox.test")
compare_means(Parasitiation_Video ~ Host, data = Daten2, method = "wilcox.test")
compare_means(Parasitiation_Video ~ Host, data = subsetTriSuz, mehtod = "wilcox.test")
compare_means(Parasitiation_Video ~ Host, data = subsetTriMel, mehtod = "wilcox.test")
compare_means(
  Parasitiation_Video ~ Host,
  data = Daten2,
  mehtod = "wilcox.test",
  group.by = "Wasp"
)
wilcox.test(Parasitiation_Video ~ Host, data = subsetTriSuz)
wilcox.test(Parasitiation_Video ~ Host, data = subsetTriMel)
wilcox.test(Parasitiation_Video ~ Wasp, data = Daten2)
```

Comparison Trichorpia suzukii / melanogaster reared

```
means <-
  ddply(Daten2,
        c("Host", "Wasp"),
        summarise,
        mean = mean(Parasitiation_Video))
means.sem <-
  ddply(
    Daten2,
    c("Host", "Wasp"),
    summarise,
    mean = mean(Parasitiation_Video),
    sem = sd(Parasitiation_Video) / sqrt(length(Parasitiation_Video))
  )
means.sem <-
  transform(means.sem, lower = mean - sem, upper = mean + sem)


means.sem_renamed <- means.sem
means.sem_renamed$Wasp <-
  gsub("T. drosophilae <melanogaster>",
       expression(paste(
         bolditalic("T. drosophilae"), bold("<melanogaster>")
       )),
       means.sem_renamed$Wasp)
means.sem_renamed$Wasp <-
  gsub("T. drosophilae <suzukii>",
```

```
      expression(paste(
        bolditalic("T. drosophilae"), bold("<suzukii>")
      )),
      means.sem_renamed$Wasp)

#Data tabel for significant levels
data_text <-
  data.frame(
    Host = c("D. suzukii", "D. suzukii"),
    mean = c(0.9, 0.9),
    lab = c("1", "2"),
    Wasp = c(means.sem_renamed$Wasp[1], means.sem_renamed$Wasp[2])
  )
data.text_renamed <- data_text
data.text_renamed$lab <-
  gsub("1",
      expression(paste(bolditalic("p adj"), bold(" = 0.04"))),
      data.text_renamed$lab)
data.text_renamed$lab <-
  gsub("2",
      expression(paste(bolditalic("p adj"), bold(" = 0.007"))),
      data.text_renamed$lab)
#boxplot
plot <-
  ggplot(means.sem_renamed, aes(x = Host, y = mean, fill = Host)) +
  geom_bar(
    stat = "identity",
    aes(colour = Host),
    position = position_dodge(width = 1),
    width = 0.6
  ) +
  #plot+
  geom_errorbar(
    aes(ymax = upper, ymin = lower),
    colour = "black",
    width = 0.2,
    position = position_dodge(width = 1)
  ) +
  geom_text(
    data = data.text_renamed,
    label = data.text_renamed$lab,
    x = 1.5,
    size = 5,
    face = "bold",
    parse = TRUE
  ) +
  facet_grid(~ Wasp, labeller = label_parsed) +
  ylim(0, 1) +
  ggplot2::labs(y = expression(bold(paste(
    "Proportion of parasitized pupae"
  )))) +
  ggplot2::theme(
    legend.position = 'none',
```

```r
    axis.title.x = element_text(size = 14),
    axis.title.y = element_text(size = 16),
    axis.text.x = element_text(face = "bold.italic", size = 16, color = "black"),
    axis.text.y = element_text(face = "bold", size = 14, color = "black"),
    strip.background = element_rect(fill = "white", colour = "black"),
    panel.background = element_rect(fill = "white", colour = "black"),
    strip.text = element_text(face = "bold", size = 16),
    axis.text = element_text(face = "bold.italic")
  ) +
  ggplot2::theme(axis.title.x = element_blank())
plot
```

```r
pdf("boxplot_Parasitization.pdf",
    width = 9,
    height = 5)
print(plot)
dev.off()
```

Building a plot of Parasitization and the pupae volume with geom_smooth for visualisation of the glm ## Plot Parasitization

```r
#suzukii_reared Trichoopria Sex wasp vs Pupeavolume ggplot_GLM
#Dataset create for p-values
data_text_plot <-
  data.frame(
    Volume = c(0.68, 0.68, 0.68, 0.72),
    Parasitiation_Video = c(0.7, 0.48, 0.53, 0.15),
    lab = c("0.89", "0.44", "0.87", "0.003"),
    Host = c("D. suzukii", "D. melanogaster", "D. suzukii", "D. melanogaster"),
    Wasp = c(
      "T. drosophilae <melanogaster>",
      "T. drosophilae <melanogaster>",
      "T. drosophilae <suzukii>",
      "T. drosophilae <suzukii>"
    )
  )

data.text_plot_renamed <- data_text_plot
data.text_plot_renamed$lab <-
  gsub("0.89",
       expression(paste(
         bolditalic("p"), bold(" = 0.89")
       )),
       data.text_plot_renamed$lab)
data.text_plot_renamed$lab <-
  gsub("0.44",
       expression(paste(
         bolditalic("p"), bold(" = 0.44")
       )),
       data.text_plot_renamed$lab)
data.text_plot_renamed$lab <-
  gsub("0.87",
       expression(paste(
         bolditalic("p"), bold(" = 0.87")
```

```
      )),
      data.text_plot_renamed$lab)
data.text_plot_renamed$lab <-
  gsub("0.003",
      expression(paste(
        bolditalic("p"), bold(" = 0.003")
      )),
      data.text_plot_renamed$lab)

plot_glm_su <-
  ggplot(aes(Volume, Parasitiation_Video, colour = Host), data = Daten2) +
  geom_smooth(method = "glm",
              method.args = list(family = "binomial"), size = 2) +
  theme_wasp() +
  ggplot2::theme(
    axis.title.x = element_blank(),
    axis.text.x = element_blank(),
    axis.ticks.x = element_blank(),
    axis.text.y = element_text(face = "bold", size = 18, color = "black"),
    axis.title.y = element_text(face = "bold", size = 18, color = "black"),
    strip.background = element_blank(),
    strip.text.x = element_blank(),
    panel.spacing = unit(2, "lines"),
    legend.position = c(0.84, 0.2),
    legend.text = element_text(face = "bold.italic", size = 14),
    legend.title = element_text(face = "bold", size = 14),
    legend.box.background = element_rect(colour = "black", size = 2)
  ) +
  facet_grid( ~ Wasp) +
  geom_text(
    data = data.text_plot_renamed,
    label = data.text_plot_renamed$lab,
    size = 4.5,
    face = "bold",
    colour = "black",
    parse = TRUE
  ) +
  labs(x = "", y = "Parasitisation")


plot_glm1 <- plot_glm_su +
  scale_y_continuous(
    breaks = seq(0,1,0.2),
    limits = c(-0.08,1.015)
  ) +
    scale_x_continuous(
      breaks = seq(0.5,5,0.5),
    limits = c(0.5,2.5)
  )
plot_glm1
```

Boxplotbuilding

Boxplot1

```
Melanogaster_boxplot <-function(Daten2_selection = NULL) {
  return(
    ggplot(data = Daten2_selection) +
      stat_boxplot(
        aes(x = Parasitiation_Video_factor, y = Volume, fill = Host),
        geom = "errorbar",
        position = position_dodge(0.9)
      ) +
      geom_boxplot(
        aes(x = Parasitiation_Video_factor, y = Volume, fill = Host),
        position = position_dodge(0.9),
        width = 0.8
      ) +
      stat_summary(
        aes(x = Parasitiation_Video_factor, y = Volume, fill = Host),
        position = position_dodge(0.9),
        fun.y = mean,
        geom = "point",
        shape = 5,
        size = 3
      ) +
      theme_wasp() +
      scale_y_continuous(breaks = seq(0.5, 5, 0.5)) +
      ggplot2::theme(aspect.ratio = 0.25,
                     panel.spacing = unit(2, "lines")) +
      coord_flip(ylim = c(
        0.5,    #min(subset_suzukii_reared_no_NA$Volumen),
        2.5       #max(subset_suzukii_reared_no_NA$Volumen)
      )) + facet_grid(~ Wasp_, labeller = label_parsed)
  )
}

Daten2_wasp_renamed <- Daten2
Daten2_wasp_renamed$Wasp_ <- factor(Daten2_wasp_renamed$Wasp,
                                    labels = c(expression(paste(
                                      bolditalic("T. drosophilae "), bold("<melanogaster>")
                                    )),
                                    expression(paste(
                                      bolditalic("T. drosophilae "), bold("<suzukii>")
                                    ))))
plot_boxplot_hatched <-
  Melanogaster_boxplot(Daten2_selection =
                         Daten2_wasp_renamed[which(Daten2$Parasitiation_Video_factor == "parasitized"),]
  ggplot2::theme(
    axis.text.x = element_blank(),
    axis.title.x = element_blank(),
    axis.ticks.x = element_blank(),
    axis.text.y = element_text(size = 18, face = "bold"),
    title = ggplot2::element_blank(),
    legend.position = "none",
    strip.background = element_rect(colour = "black", fill = "white"),
```

```
    strip.text.x = element_text(face = "bold", size = 15)
  )


# Lower boxplot holding the title for all 3 x axis (2x box + GLM)
# Text lable placed by labs(...)
plot_boxplot_hatched_not <-
  Melanogaster_boxplot(Daten2_selection =
                        Daten2_wasp_renamed[which(Daten2$Parasitiation_Video_factor == "not parasitize
  ggplot2::labs(y = expression(bold(paste(
    "Pupae Volume [", mm ^ 3, "]"
  ))), x = "") +
  theme(axis.text.y = element_text(size = 18, face = "bold"),
        axis.text.x = element_text(size = 18, face = "bold")) +
  ggplot2::theme(
    strip.background = element_blank(),
    strip.text.x = element_blank(),
    legend.position = "none"
  )
 #combining both boxplots in one page (just for showing)
cowplot::plot_grid(
  plot_boxplot_hatched,
  plot_boxplot_hatched_not,
  align = c("v"),
  nrow = 2
)
```

Mulitple plot suzukii reared Trichopria

```
plot_aligned <-
  cowplot::align_plots(
    plot_boxplot_hatched,
    plot_glm1,
    plot_boxplot_hatched_not,
    align = c("v"),
    axis = c("lr")
  )

plot_suzukii_reared_volume_vs_sex <- cowplot::ggdraw() +
  cowplot::draw_plot(plot_aligned[[2]], 0, 0.14, 1, 0.7) +
  cowplot::draw_plot(plot_aligned[[1]], 0, 0.4, 1, 1) +
  cowplot::draw_plot(plot_aligned[[3]], 0,-0.4, 1, 1)
plot_suzukii_reared_volume_vs_sex
```

```
pdf("plot_Parasitization.pdf",
    width = 9,
    height = 7)
print(plot_suzukii_reared_volume_vs_sex)
dev.off()
```

Parasitization Success

GLM Parasitization Success

```
#sink('GLM-Geschlecht_Wespe_virilis-output.txt')
m11 <-
  glmer(
    Parasitation_succes ~  Host * Volume + Wasp + (1 |
                                                     Repetition),
    data = Daten2,
    family = binomial
  )
r.squaredGLMM(m11)
summary(m11)
Anova(m11)
summary(glht(m11, mcp(Host = "Tukey")))
summary(glht(m11, mcp(Wasp = "Tukey")))
#sink()


m13 <-
  glmer(
    Parasitation_succes ~  Host * Volume + (1 |
                                              Repetition),
    data = subsetTriSuz,
    family = binomial
  )
r.squaredGLMM(m13)
summary(m13)
Anova(m13)
summary(glht(m13, mcp(Host = "Tukey")))
#sink()


m12 <-
  glmer(
    Parasitation_succes ~  Host * Volume + (1 |
                                              Repetition),
    data = subsetTriMel ,
    family = binomial
  )
r.squaredGLMM(m12)
summary(m12)
Anova(m12)
summary(glht(m12, mcp(Host = "Tukey")))
#sink()

## Subsubsets!!!

m14 <-
  glmer(Parasitation_succes ~ Volume + (1 |
                                          Repetition),
        data = subsetMelTriMel,
        family = binomial)
```

```
## boundary (singular) fit: see ?isSingular
```

```r
r.squaredGLMM(m14)
summary(m14)
Anova(m14)
#sink()
m15 <-
  glmer(Parasitation_succes ~ Volume + (1 |
                                          Repetition),
        data = subsetMelTriSuz,
        family = binomial)
r.squaredGLMM(m15)
summary(m15)
Anova(m15)
#sink()

m16 <-
  glmer(Parasitation_succes ~ Volume + (1 |
                                          Repetition),
        data = subsetSuzTriSuz,
        family = binomial)
r.squaredGLMM(m16)
summary(m16)
Anova(m16)
#sink()

m17 <-
  glmer(Parasitation_succes ~ Volume + (1 |
                                          Repetition),
        data = subsetSuzTriMel,
        family = binomial)
r.squaredGLMM(m17)
summary(m17)
Anova(m17)
#sink()
```

Plot

```r
data_text_plot2 <-
  data.frame(
    Volume = c(2.37, 2.37, 2.3, 2.3),
    Parasitation_succes = c(0.1, 0.35, 0.09, 0.52),
    lab = c("0.07", "0.02", "0.10", "0.7"),
    Host = c("D. suzukii", "D. melanogaster", "D. suzukii", "D. melanogaster"),
    Wasp = c(
      "T. drosophilae <melanogaster>",
      "T. drosophilae <melanogaster>",
      "T. drosophilae <suzukii>",
      "T. drosophilae <suzukii>"
    )
  )

data.text_plot2_renamed <- data_text_plot2
data.text_plot2_renamed$lab <-
```

```r
  gsub("0.07",
       expression(paste(bolditalic("p"), bold(" = 0.07"))),
       data.text_plot2_renamed$lab)
data.text_plot2_renamed$lab <-
  gsub("0.02",
       expression(paste(bolditalic("p"), bold(" = 0.02"))),
       data.text_plot2_renamed$lab)
data.text_plot2_renamed$lab <-
  gsub("0.10",
       expression(paste(bolditalic("p"), bold(" = 0.10"))),
       data.text_plot2_renamed$lab)
data.text_plot2_renamed$lab <-
  gsub("0.7",
       expression(paste(bolditalic("p"), bold(" = 0.7"))),
       data.text_plot2_renamed$lab)

plot_glm_su <-
  ggplot(aes(Volume, Parasitation_succes, colour = Host), data = Daten2) +
  geom_smooth(method = "glm",
              method.args = list(family = "binomial"), size = 2) +
  theme_wasp() +
  ggplot2::theme(
    axis.title.x = element_blank(),
    axis.text.x = element_blank(),
    axis.ticks.x = element_blank(),
    axis.text.y = element_text(face = "bold", size = 18, color = "black"),
    axis.title.y = element_text(face = "bold", size = 18, color = "black"),
    strip.background = element_blank(),
    strip.text.x = element_blank(),
    panel.spacing = unit(2, "lines"),
    legend.position = c(0.15, 0.22),
    legend.text = element_text(face = "bold.italic", size = 14),
    legend.title = element_text(face = "bold", size = 14),
    #legend.box.background = element_rect(colour = "black", size = 2),
    legend.background = element_rect(colour = "transparent", fill = "transparent")
  ) +
  facet_grid(~ Wasp) +
  geom_text(
    data = data.text_plot2_renamed,
    label = data.text_plot2_renamed$lab,
    size = 4.5,
    face = "bold",
    colour = "black",
    parse = TRUE
  ) +
  # Removing x lable already placed by plot_boxplot_hatched_not ggplot_GLM
  labs(x = "", y = "Parasitisation Success")

plot_glm11 <- plot_glm_su +
  scale_y_continuous(breaks = seq(0, 1, 0.2),
                     limits = c(-0.08, 1)) +
  scale_x_continuous(breaks = seq(0.5, 5, 0.5),
                     limits = c(0.5, 2.5))
```

```
#pdf("random_effect_plot_virilis_volume_vs_sex.pdf", width = 9, height = 7)
plot_glm11
```

Boxplotbuilding

Boxplot1

```
Melanogaster_boxplot2 <- function(Daten2_selection = NULL) {
  return(
    ggplot(data = Daten2_selection) +
      stat_boxplot(
        aes(x = Parasitiation_success_factor, y = Volume, fill = Host),
        geom = "errorbar",
        position = position_dodge(0.9)
      ) +
      geom_boxplot(
        aes(x = Parasitiation_success_factor, y = Volume, fill = Host),
        position = position_dodge(0.9),
        width = 0.8
      ) +
      stat_summary(
        aes(x = Parasitiation_success_factor, y = Volume, fill = Host),
        position = position_dodge(0.9),
        fun.y = mean,
        geom = "point",
        shape = 5,
        size = 3
      ) +
      theme_wasp() +
      scale_y_continuous(breaks = seq(0.5, 5, 0.5)) +
      ggplot2::theme(
        aspect.ratio = 0.2,
        panel.spacing = unit(2, "lines")
      ) +
      coord_flip(ylim = c(
        0.5,     #min(subset_suzukii_reared_no_NA$Volumen),
        2.5        #max(subset_suzukii_reared_no_NA$Volumen)
      )) +
      facet_grid( ~ Wasp_, labeller = label_parsed)
  )
}
plot_boxplot_hatched1 <-
  Melanogaster_boxplot2(Daten2_selection =
                          Daten2_wasp_renamed[which(Daten2$Parasitiation_success_factor == "success"), ]
  ggplot2::theme(
    axis.text.x = element_blank(),
    axis.title.x = element_blank(),
    axis.ticks.x = element_blank(),
    axis.text.y = element_text(size = 18, face = "bold"),
    title = ggplot2::element_blank(),
    legend.position = "none",
    strip.background = element_rect(colour = "black", fill = "white"),
    strip.text.x = element_text(face = "bold", size = 15)
```

```
  )
# Lower boxplot holding the title for all 3 x axis (2x box + GLM)
# Text lable placed by labs(...)
plot_boxplot_hatched_not1 <-
  Melanogaster_boxplot2(Daten2_selection =
                          Daten2_wasp_renamed[which(Daten2$Parasitiation_success_factor == "no success")
  ggplot2::labs(y = expression(bold(paste(
    "Pupae Volume [", mm ^ 3, "]"
  ))), x = "") +
  theme(axis.text.y = element_text(size = 18, face = "bold"),
        axis.text.x = element_text(size = 18, face = "bold")) +
  ggplot2::theme(
    strip.background = element_blank(),
    strip.text.x = element_blank(),
    legend.position = "none"
  )
#combining both boxplots in one page (just for showing)
cowplot::plot_grid(
  plot_boxplot_hatched1,
  plot_boxplot_hatched_not1,
  align = c("v"),
  nrow = 2
)
```

Mulitple plot suzukii reared Trichopria

```
plot_aligned1 <-
  cowplot::align_plots(
    plot_boxplot_hatched1,
    plot_glm11,
    plot_boxplot_hatched_not1,
    align = c("v"),
    axis = c("lr")
  )
plot_suzukii_reared_volume_vs_sex <- cowplot::ggdraw() +
  cowplot::draw_plot(plot_aligned1[[2]], 0, 0.14, 1, 0.7) +
  cowplot::draw_plot(plot_aligned1[[1]], 0, 0.4, 1, 1) +
  cowplot::draw_plot(plot_aligned1[[3]], 0,-0.4, 1, 1)
plot_suzukii_reared_volume_vs_sex
```

```
pdf("plot_Parasitization_sucess.pdf",
    width = 9,
    height = 7)
print(plot_suzukii_reared_volume_vs_sex)
dev.off()
```

Significanc test Sucess of Parasitization

Compare_means with wilcox.test

```
compare_means(Parasitation_succes ~ Wasp, data = Daten2, method = "wilcox.test")
compare_means(Parasitation_succes ~ Host, data = Daten2, method = "wilcox.test")
compare_means(Parasitation_succes ~ Host, data = subsetTriSuz, mehtod = "wilcox.test")
```

```r
compare_means(Parasitation_succes ~ Host, data = subsetTriMel, mehtod = "wilcox.test")
compare_means(
  Parasitation_succes ~ Host,
  data = Daten2,
  mehtod = "wilcox.test",
  group.by = "Wasp"
)
wilcox.test(Parasitation_succes ~ Host, data = subsetTriSuz)
wilcox.test(Parasitation_succes ~ Host, data = subsetTriMel)
wilcox.test(Parasitation_succes ~ Wasp, data = Daten2)
```

Box plot succes of Parasitism

```r
#DataTabel for means
means2 <-
  ddply(Daten2,
        c("Host", "Wasp"),
        summarise,
        mean = mean(Parasitation_succes, na.rm = T))
means.sem2 <-
  ddply(
    Daten2,
    c("Host", "Wasp"),
    summarise,
    mean = mean(Parasitation_succes, na.rm = T),
    sem = sd(Parasitation_succes, na.rm = T) / sqrt(length(Parasitation_succes))
  )
means.sem2 <-
  transform(means.sem2, lower = mean - sem, upper = mean + sem)
#Data tabel for significant levels
data_text <-
  data.frame(
    Host = c("D. suzukii", "D. suzukii"),
    mean = c(0.9, 0.9),
    lab = c("1", "2"),
    Wasp = c("T. drosophilae <melanogaster>", "T. drosophilae <suzukii>")
  )
means.sem2$Wasp <-
  gsub("T. drosophilae <melanogaster>",
       expression(paste(
         bolditalic("T. drosophilae "), bold("<melanogaster>")
       )),
       means.sem2$Wasp)
means.sem2$Wasp <-
  gsub("T. drosophilae <suzukii>",
       expression(paste(
         bolditalic("T. drosophilae "), bold("<suzukii>")
       )),
       means.sem2$Wasp)
data_text$Wasp <-
  gsub("T. drosophilae <melanogaster>",
       expression(paste(
         bolditalic("T. drosophilae "), bold("<melanogaster>")
```

```
      )),
      data_text$Wasp)
data_text$Wasp <-
  gsub("T. drosophilae <suzukii>", expression(paste(
    bolditalic("T. drosophilae "), bold("<suzukii>")
  )), data_text$Wasp)

data.text_renamed <- data_text
data.text_renamed$lab <-
  gsub("1",
      expression(paste(bolditalic("p adj"), bold(" = 0.16"))),
      data.text_renamed$lab)
data.text_renamed$lab <-
  gsub("2",
      expression(paste(bolditalic("p adj"), bold(" = 0.18"))),
      data.text_renamed$lab)
#boxplot
plot <- ggplot(means.sem2, aes(x = Host, y = mean, fill = Host)) +
  geom_bar(
    stat = "identity",
    aes(colour = Host),
    position = position_dodge(width = 1),
    width = 0.6
  ) +
  #plot+
  geom_errorbar(
    aes(ymax = upper, ymin = lower),
    colour = "black",
    width = 0.2,
    position = position_dodge(width = 1)
  ) +
  geom_text(
    data = data.text_renamed,
    label = data.text_renamed$lab,
    x = 1.5,
    size = 5,
    face = "bold",
    parse = TRUE
  ) +
  facet_grid(~ Wasp, labeller = label_parsed) +
  ylim(0, 1) +
  ggplot2::labs(y = expression(bold(paste(
    "Successful Parasitization"
  )))) +
  ggplot2::theme(
    legend.position = "none",
    legend.title = element_text(face = "bold", size = 9),
    legend.text = element_text(face = "bold.italic", size = 8),
    legend.background = element_rect(
      size = 0.4,
      linetype = "solid",
      colour = "black"
    ),
```

```
    axis.title.x = element_text(size = 14),
    axis.title.y = element_text(size = 16),
    axis.text.x = element_text(face = "bold.italic", size = 16, color = "black"),
    axis.text.y = element_text(face = "bold", size = 14, colour = "black"),
    strip.background = element_rect(fill = "white", colour = "black"),
    panel.background = element_rect(fill = "white", colour = "black"),
    strip.text = element_text(face = "bold", size = 16),
    axis.text = element_text(face = "bold.italic")
  ) +
  ggplot2::theme(axis.title.x = element_blank())
plot
```

```
pdf("boxplot_sucess_parasitism.pdf",
    width = 9,
    height = 5)
print(plot)
dev.off()
```

Significanc test FLY Sucess of Parasitization

```
compare_means(Fly_success_parasitization ~ Wasp,
              data = Daten2,
              method = "wilcox.test")
compare_means(Fly_success_parasitization ~ Host,
              data = Daten2,
              method = "wilcox.test")
compare_means(Fly_success_parasitization ~ Host,
              data = subsetTriSuz,
              mehtod = "wilcox.test")
compare_means(Fly_success_parasitization ~ Host,
              data = subsetTriMel,
              mehtod = "wilcox.test")
compare_means(
  Fly_success_parasitization ~ Host,
  data = Daten2,
  mehtod = "wilcox.test",
  group.by = "Wasp"
)
wilcox.test(Fly_success_parasitization ~ Host, data = subsetTriSuz)
wilcox.test(Fly_success_parasitization ~ Host, data = subsetTriMel)
wilcox.test(Fly_success_parasitization ~ Wasp, data = Daten2)
```

Box plot FLY succes of Parasitism

Probablilty that a fly overcomes a parasitizaiton

```
#DataTabel for means
means3 <-
  ddply(Daten2,
        c("Host", "Wasp"),
        summarise,
        mean = mean(Fly_success_parasitization, na.rm = T))
means.sem3 <-
```

```
  ddply(
    Daten2,
    c("Host", "Wasp"),
    summarise,
    mean = mean(Fly_success_parasitization, na.rm = T),
    sem = sd(Fly_success_parasitization, na.rm = T) / sqrt(length(Fly_success_parasitization))
  )
means.sem3 <-
  transform(means.sem3, lower = mean - sem, upper = mean + sem)


#Data tabel for significant levels
data_text2 <-
  data.frame(
    Host = c("D. suzukii", "D. suzukii"),
    mean = c(0.45, 0.45),
    lab = c("1", "2"),
    Wasp = c("T. drosophilae <melanogaster>", "T. drosophilae <suzukii>")
  )

means.sem3$Wasp <-
  gsub("T. drosophilae <melanogaster>",
       expression(paste(
         bolditalic("T. drosophilae "), bold("<melanogaster>")
       )),
       means.sem3$Wasp)
means.sem3$Wasp <-
  gsub("T. drosophilae <suzukii>",
       expression(paste(
         bolditalic("T. drosophilae "), bold("<suzukii>")
       )),
       means.sem3$Wasp)
data_text2$Wasp <-
  gsub("T. drosophilae <melanogaster>",
       expression(paste(
         bolditalic("T. drosophilae "), bold("<melanogaster>")
       )),
       data_text2$Wasp)
data_text2$Wasp <-
  gsub("T. drosophilae <suzukii>",
       expression(paste(
         bolditalic("T. drosophilae "), bold("<suzukii>")
       )),
       data_text2$Wasp)

data.text_renamed2 <- data_text2
data.text_renamed2$lab <-
  gsub("1",
       expression(paste(bolditalic("p adj"), bold(" = <0.001"))),
       data.text_renamed2$lab)
data.text_renamed2$lab <-
  gsub("2",
       expression(paste(bolditalic("p adj"), bold(" = <0.001"))),
```

```r
      data.text_renamed2$lab)


#boxplot
plot2 <- ggplot(means.sem3, aes(x = Host, y = mean, fill = Host)) +
  geom_bar(
    stat = "identity",
    aes(colour = Host),
    position = position_dodge(width = 1),
    width = 0.6
  ) +
  #plot+
  geom_errorbar(
    aes(ymax = upper, ymin = lower),
    colour = "black",
    width = 0.2,
    position = position_dodge(width = 1)
  ) +
  geom_text(
    data = data.text_renamed2,
    label = data.text_renamed2$lab,
    x = 1.5,
    size = 5,
    face = "bold",
    parse = TRUE
  ) +
  facet_grid(~ Wasp, labeller = label_parsed) +
  ylim(0, 1) +
  ggplot2::labs(y = expression(bold(
    paste("Proportion of hatched hosts when parasitized")
  ))) +
  ggplot2::theme(
    legend.position = "none",
    legend.title = element_text(face = "bold", size = 9),
    legend.text = element_text(face = "bold.italic", size = 8),
    legend.background = element_rect(
      size = 0.4,
      linetype = "solid",
      colour = "black"
    ),
    axis.title.x = element_text(size = 14),
    axis.title.y = element_text(size = 16),
    axis.text.x = element_text(face = "bold.italic", size = 16, color = "black"),
    axis.text.y = element_text(face = "bold", size = 14, colour = "black"),
    strip.background = element_rect(fill = "white", colour = "black"),
    panel.background = element_rect(fill = "white", colour = "black"),
    strip.text = element_text(face = "bold", size = 16),
    axis.text = element_text(face = "bold.italic")
  ) +
  scale_y_continuous(breaks = seq(0, 1, 0.1),
                     limits = c(0, 0.5)) +
  ggplot2::theme(axis.title.x = element_blank())
```

```
## Scale for 'y' is already present. Adding another scale for 'y', which
```

```
## will replace the existing scale.
```

```
plot2
```

```
pdf("boxplot_FLY_sucess_parasitism.pdf",
    width = 9,
    height = 5)
print(plot2)
dev.off()
```

```
compare_means(Fly_success_parasitization ~ Wasp,
              data = Daten2,
              method = "wilcox.test")
compare_means(Fly_success_parasitization ~ Host,
              data = Daten2,
              method = "wilcox.test")
compare_means(Fly_success_parasitization ~ Host,
              data = subsetTriSuz,
              mehtod = "wilcox.test")
compare_means(Fly_success_parasitization ~ Host,
              data = subsetTriMel,
              mehtod = "wilcox.test")
compare_means(
  Fly_success_parasitization ~ Host,
  data = Daten2,
  mehtod = "wilcox.test",
  group.by = "Wasp"
)
```

Sex Ratio D. suzukii

```
Daten_suz_sex <-
  read_excel("suzukii Tabelle.xlsx",
             sheet = "Tabelle1",
             na = "NA")
#subset_plot1
Daten_suz_sex$Wespe_Geschlecht_String <-
  str_replace_all(as.character(Daten_suz_sex$Wasp_sex), "0", "male")
Daten_suz_sex$Wespe_Geschlecht_String <-
  str_replace_all(as.character(Daten_suz_sex$Wespe_Geschlecht_String),
                  "1",
                  "female")

subset_No_control_No_NA <- subset(
  Daten_suz_sex,
  Wespe_Geschlecht_String == "male" |
    Wespe_Geschlecht_String == "female"
)
subset_No_control_No_NA$Wespe_Geschlecht_Factor <-
  as.factor(subset_No_control_No_NA$Wespe_Geschlecht_String)
is.factor(subset_No_control_No_NA$Wespe_Geschlecht_Factor)

Daten_suz_sex$Repetition <- as.factor(Daten_suz_sex$Repetition)
```

```r
is.factor(subset_No_control_No_NA$Wespe_Geschlecht_Factor)
is.factor(Daten_suz_sex$Repetition)
```

##GLM

```r
# GLM Geschlecht_Schlupf_Wespe abhänig vom Volumen der Fliege
sink('GLM-Geschlecht_Wespe_suz-output.txt')
m1 <-
  glmer(Wasp_sex ~ Volume +  (1 |
                                 Repetition),
        data = Daten_suz_sex,
        family = binomial)
r.squaredGLMM(m1)
summary(m1)
Anova(m1)
sink()
```

##Plot building

```r
#suzukii_reared Trichoopria Sex wasp vs Pupeavolume ggplot_GLM
plot_glm_su <-
  ggplot(aes(Volume, Wasp_sex), data = subset_No_control_No_NA) +
  geom_smooth(method = "glm",
              method.args = list(family = "binomial")) +
  annotate(
    "text",
    x = 3.2,
    y = 0.55,
    label = "paste(bolditalic(p),bold( \" = 0.016\"))",
    size = 4.5,
    parse = TRUE
  ) +
  theme_wasp() +
  ggplot2::theme(
    axis.title.x = element_blank(),
    axis.text.x = element_blank(),
    axis.ticks.x = element_blank()
  ) +
  # Removing x lable already placed by plot_boxplot_hatched_not ggplot_GLM
  labs(x = "", y = "Sex Wasp")

plot_glm1 <- plot_glm_su +
  scale_y_continuous(breaks = seq(0, 1, 0.2),
                     limits = c(-0.08, 1)) +
  scale_x_continuous(breaks = seq(1, 3.5, 0.5),
                     limits = c(1, 3.5))
plot_glm1
```

##Boxplotbuilding

```r
Melanogaster_boxplot <-
  function(subset_No_control_No_NA_selection = NULL) {
    return(
      ggplot(data = subset_No_control_No_NA_selection) +
        stat_boxplot(aes(x = Wespe_Geschlecht_Factor, y = Volume),
                     geom = "errorbar",) +
```

```
        geom_boxplot(
          aes(x = Wespe_Geschlecht_Factor, y = Volume),
          position = position_dodge(0.9),
          width = 1
        ) +
        stat_summary(
          aes(x = Wespe_Geschlecht_Factor, y = Volume),
          fun.y = mean,
          geom = "point",
          shape = 23,
          size = 4
        ) +
        theme_wasp() +
        scale_y_continuous(breaks = seq(1, 3.5, 0.5)) +
        ggplot2::theme(aspect.ratio = 0.1) +
      coord_flip(ylim = c(
        1,    #min(subset_suzukii_reared_no_NA$Volumen),
        3.5       #max(subset_suzukii_reared_no_NA$Volumen)
      ))
    )
  }
plot_boxplot_hatched <-
  Melanogaster_boxplot(subset_No_control_No_NA_selection = subset_No_control_No_NA[which(subset_No_cont


  ggplot2::theme(
    axis.text.x = element_blank(),
    axis.title.x = element_blank(),
    axis.ticks.x = element_blank(),
    title = ggplot2::element_blank()
  )

plot_boxplot_hatched_not <-
  Melanogaster_boxplot(subset_No_control_No_NA_selection = subset_No_control_No_NA[which(subset_No_cont
  ggplot2::labs(y = expression(bold(paste(
    "Pupae Volume [", mm ^ 3, "]"
  ))), x = "") +
  theme(axis.title.x = element_text(size = 18))

# combining both boxplots in one page (just for showing)
cowplot::plot_grid(
  plot_boxplot_hatched,
  plot_boxplot_hatched_not,
  align = c("v"),
  nrow = 2
)
```

Mulitple plot

```
plot_aligned <-
  cowplot::align_plots(
    plot_boxplot_hatched,
    plot_glm1,
```

```
    plot_boxplot_hatched_not,
    align = c("v"),
    axis = c("lr")
  )

plot_suzukii_reared_volume_vs_sex <- cowplot::ggdraw() +
  cowplot::draw_plot(plot_aligned[[2]], 0, 0.15, 1, 0.7) +
  cowplot::draw_plot(plot_aligned[[1]], 0, 0.4, 1, 1) +
  cowplot::draw_plot(plot_aligned[[3]], 0,-0.4, 1, 1)
plot_suzukii_reared_volume_vs_sex
```

```
pdf("plot_suzukii_volume_vs_sex.pdf",
    width = 6,
    height = 6)
print(plot_suzukii_reared_volume_vs_sex)
dev.off()
```

Sex ratio D. melanogaster

```
Data_sex_mel <-
  read_excel("Melanogaster Tabelle.xlsx",
             sheet = "Tabelle1",
             na = "NA")
#subset_plot1
Data_sex_mel$Wespe_Geschlecht_String <-
  str_replace_all(as.character(Data_sex_mel$Wasp_sex), "0", "male")
Data_sex_mel$Wespe_Geschlecht_String <-
  str_replace_all(as.character(Data_sex_mel$Wespe_Geschlecht_String),
                  "1",
                  "female")
subset_No_control_No_NA <- subset(Data_sex_mel,
                                  Wespe_Geschlecht_String == "male" |
                                    Wespe_Geschlecht_String == "female")
subset_No_control_No_NA$Wespe_Geschlecht_Factor <-
  as.factor(subset_No_control_No_NA$Wespe_Geschlecht_String)
is.factor(subset_No_control_No_NA$Wespe_Geschlecht_Factor)
Data_sex_mel$Repetition <- as.factor(Data_sex_mel$Repetition)

is.factor(subset_No_control_No_NA$Wespe_Geschlecht_Factor)
is.factor(Data_sex_mel$Repetition)
```

##GLM

```
# GLM Geschlecht_Schlupf_Wespe abhänig vom Volumen der Fliege
sink('GLM-Geschlecht_Wespe_melanogaster-output.txt')
m1 <-
  glmer(Wasp_sex ~ Volume +  (1 |
                                Repetition),
        data = Data_sex_mel,
        family = binomial)
r.squaredGLMM(m1)
summary(m1)
Anova(m1)
```

```r
sink()
```

Plot building

```r
#suzukii_reared Trichoopria Sex wasp vs Pupeavolume ggplot_GLM
plot_glm_su <-
  ggplot(aes(Volume, Wasp_sex), data = subset_No_control_No_NA) +
  geom_smooth(method = "glm",
              method.args = list(family = "binomial")) +
  annotate(
    "text",
    x = 2.2,
    y = 0.7,
    label = "paste(bolditalic(p),bold( \" = 0.0004\"))",
    size = 4.5,
    parse = TRUE
  ) +
  theme_wasp() +
  ggplot2::theme(
    axis.title.x = element_blank(),
    axis.text.x = element_blank(),
    axis.ticks.x = element_blank()
  ) +
  # Removing x lable already placed by plot_boxplot_hatched_not ggplot_GLM
  labs(x = "", y = "Sex Wasp")

plot_glm1 <- plot_glm_su +
  scale_y_continuous(breaks = seq(0, 1, 0.2),
                     limits = c(-0.08, 1)) +
  scale_x_continuous(breaks = seq(0.5, 2.5, 0.5),
                     limits = c(0.5, 2.5))
plot_glm1
```

Boxplotbuilding

```r
Melanogaster_boxplot <-function(subset_No_control_No_NA_selection = NULL) {
  return(
    ggplot(data = subset_No_control_No_NA_selection) +
      stat_boxplot(aes(x = Wespe_Geschlecht_Factor, y = Volume),
                   geom = "errorbar",) +
      geom_boxplot(
        aes(x = Wespe_Geschlecht_Factor, y = Volume),
        position = position_dodge(0.9),
        width = 1
      ) +
      stat_summary(
        aes(x = Wespe_Geschlecht_Factor, y = Volume),
        fun.y = mean,
        geom = "point",
        shape = 23,
        size = 4
      ) +
```

```
      theme_wasp() +
      scale_y_continuous(breaks = seq(0.5, 2.5, 0.5)) +
      ggplot2::theme(aspect.ratio = 0.1) +
      coord_flip(ylim = c(
        0.5,    #min(subset_suzukii_reared_no_NA$Volumen),
        2.5       #max(subset_suzukii_reared_no_NA$Volumen)
      ))
  )
}
#select ony hatched rows [ROW,col], which outputs every row number matching
plot_boxplot_hatched <-
  Melanogaster_boxplot(subset_No_control_No_NA_selection = subset_No_control_No_NA[which(subset_No_cont

  ggplot2::theme(
    axis.text.x = element_blank(),
    axis.title.x = element_blank(),
    axis.ticks.x = element_blank(),
    title = ggplot2::element_blank()
  )

plot_boxplot_hatched_not <-
  Melanogaster_boxplot(subset_No_control_No_NA_selection = subset_No_control_No_NA[which(subset_No_cont
  ggplot2::labs(y = expression(bold(paste(
    "Pupae Volume [", mm ^ 3, "]"
  ))), x = "") +
  theme(axis.title.x = element_text(size = 18))


# combining both boxplots in one page (just for showing)
cowplot::plot_grid(
  plot_boxplot_hatched,
  plot_boxplot_hatched_not,
  align = c("v"),
  nrow = 2
)
```

Mulitple plot

```
plot_aligned <-
  cowplot::align_plots(
    plot_boxplot_hatched,
    plot_glm1,
    plot_boxplot_hatched_not,
    align = c("v"),
    axis = c("lr")
  )

plot_melanogaster_reared_volume_vs_sex <- cowplot::ggdraw() +
  cowplot::draw_plot(plot_aligned[[2]], 0, 0.15, 1, 0.7 ) +
  cowplot::draw_plot(plot_aligned[[1]], 0, 0.4, 1, 1) +
  cowplot::draw_plot(plot_aligned[[3]], 0,-0.4, 1, 1 )
plot_melanogaster_reared_volume_vs_sex
```

```r
pdf("plot_melanogaster_volume_vs_sex.pdf",
    width = 6,
    height = 6)
print(plot_melanogaster_reared_volume_vs_sex)
dev.off()
```

Sex Ratio Choice expirement

```r
Data_mel_suz <-
  read_excel("Suzu melan Tabelle.xlsx",
             sheet = "Tabelle1",
             na = "NA")
#subset creation
Data_mel_suz$Wasp_sex_String <-
  str_replace_all(as.character(Data_mel_suz$Wasp_sex), "0", "male")
Data_mel_suz$Wasp_sex_String <-
  str_replace_all(as.character(Data_mel_suz$Wasp_sex_String), "1", "female")
Data_mel_suz <- subset(Data_mel_suz,
                       Wasp_sex_String == "male" | Wasp_sex_String == "female")
Data_mel_suz$Wasp_sex_Factor <-
  as.factor(Data_mel_suz$Wasp_sex_String)
is.factor(Data_mel_suz$Wasp_sex_Factor)
subset_Mel <- subset(Data_mel_suz, Pupae == 'Melanogaster')
subset_Suz <- subset(Data_mel_suz, Pupae == 'Suzukii')
Data_mel_suz$Repetition <- as.factor(Data_mel_suz$Repetition)
is.factor(Data_mel_suz$Wasp_sex_Factor)
is.factor(Data_mel_suz$Repetition)
```

GLM

```r
# GLM Sex wasp to Volume of the fly pupae
#sink('GLM-Geschlecht_Wespe_melanogaster-output.txt')
m1 <-
  glmer(Wasp_sex ~ Volume * Pupae +  (1 |
                                        Repetition),
        data = Data_mel_suz,
        family = binomial)
r.squaredGLMM(m1)
summary(m1)
Anova(m1)
summary(glht(m1, mcp(Pupae = "Tukey")))
#sink()

#sink('GLM-Geschlecht_Wespe_melanogaster-output.txt')
m2 <-
  glmer(Wasp_sex ~ Volume +  (1 |
                                Repetition),
        data = subset_Suz,
        family = binomial)
```

```
## boundary (singular) fit: see ?isSingular
```

```r
r.squaredGLMM(m2)
```

```
## boundary (singular) fit: see ?isSingular
```

```r
summary(m2)
Anova(m2)
#sink()

#sink('GLM-Geschlecht_Wespe_melanogaster-output.txt')
m3 <-
  glmer(Wasp_sex ~ Volume +  (1 |
                                Repetition),
        data = subset_Mel,
        family = binomial)
r.squaredGLMM(m3)
summary(m3)
Anova(m3)
#sink()
```

Plot building

```r
#dataset create for p-values
data_text_plot <-
  data.frame(
    Volume = c(0.8, 0.8),
    Wasp_sex = c(0.33, 0.67),
    lab = c("0.49", "0.2")
  )
data.text_plot_renamed <- data_text_plot
data.text_plot_renamed$lab <-
  gsub("0.49",
       expression(paste(bolditalic("p"), bold(" = 0.49"))),
       data.text_plot_renamed$lab)
data.text_plot_renamed$lab <-
  gsub("0.2",
       expression(paste(bolditalic("p"), bold(" = 0.2"))),
       data.text_plot_renamed$lab)
#suzukii_reared Trichoopria Sex wasp vs Pupeavolume ggplot_GLM
plot_glm_su <-
  ggplot(aes(Volume, Wasp_sex, colour = Pupae), data = Data_mel_suz) +
  geom_smooth(method = "glm",
              method.args = list(family = "binomial"), size = 2) +
  geom_text(
    data = data.text_plot_renamed,
    label = data.text_plot_renamed$lab,
    size = 5,
    face = "bold",
    colour = "black",
    parse = TRUE
  ) +
  theme_wasp() +
  ggplot2::theme(
    axis.title.x = element_blank(),
    axis.text.x = element_blank(),
```

```
    axis.ticks.x = element_blank(),
    legend.position = c(0.88, 0.84),
    legend.text = element_text(face = "bold.italic", size = 14),
    legend.title = element_text(face = "bold", size = 14),
    legend.box.background = element_rect(colour = "black", size = 2),
    #scale_fill_discrete(name = "Host")
  ) +
  # Removing x lable already placed by plot_boxplot_hatched_not ggplot_GLM
  labs(x = "", y = "Sex Wasp")

plot_glm1 <- plot_glm_su +
  scale_y_continuous(breaks = seq(0, 1, 0.2),
                     limits = c(-0.08, 1)) +
  scale_x_continuous(breaks = seq(0.5, 2.5, 0.5),
                     limits = c(0.5, 3.1))
plot_glm1
```

Boxplotbuilding

```
Melanogaster_boxplot <- function(Data_mel_suz_selection = NULL) {
  return(
    ggplot(data = Data_mel_suz_selection) +
      stat_boxplot(
        aes(x = Wasp_sex_Factor, y = Volume, fill = Pupae),
        geom = "errorbar",
        position = position_dodge(0.9)
      ) +
      geom_boxplot(
        aes(x = Wasp_sex_Factor, y = Volume, fill = Pupae),
        position = position_dodge(0.9),
        width = 0.8
      ) +
      stat_summary(
        aes(x = Wasp_sex_Factor, y = Volume, fill = Pupae),
        position = position_dodge(0.9),
        fun.y = mean,
        geom = "point",
        shape = 5,
        size = 4
      ) +
      theme_wasp() +
      scale_y_continuous(breaks = seq(0.5, 5, 0.5)) +

      ggplot2::theme(
        aspect.ratio = 0.125,
        panel.spacing = unit(2, "lines")
      ) +
      coord_flip(ylim = c(
        0.5,    #min(subset_suzukii_reared_no_NA$Volume),
        3.1       #max(subset_suzukii_reared_no_NA$Volume)
      ))
  )
}
```

```
plot_boxplot_hatched <-
  Melanogaster_boxplot(Data_mel_suz_selection = Data_mel_suz[which(Data_mel_suz$Wasp_sex_Factor == "fema
  ggplot2::theme(
    axis.text.x = element_blank(),
    axis.title.x = element_blank(),
    axis.ticks.x = element_blank(),
    title = ggplot2::element_blank()
  ) +
  ggplot2::theme(
    strip.background = element_blank(),
    strip.text.x = element_blank(),
    legend.position = "none"
  )
# Lower boxplot holding the title for all 3 x axis (2x box + GLM)
# Text lable placed by labs(...)
plot_boxplot_hatched_not <-
  Melanogaster_boxplot(Data_mel_suz_selection = Data_mel_suz[which(Data_mel_suz$Wasp_sex_Factor == "male
  ggplot2::labs(y = expression(bold(paste(
    "Pupae Volume [", mm ^ 3, "]"
  ))), x = "") +
  theme(axis.title.x = element_text(size = 18)) +
  ggplot2::theme(
    strip.background = element_blank(),
    strip.text.x = element_blank(),
    legend.position = "none"
  )
# combining both boxplots in one page (just to see)
cowplot::plot_grid(
  plot_boxplot_hatched,
  plot_boxplot_hatched_not,
  align = c("v"),
  nrow = 2
)
```

Mulitple plot

```
plot_aligned <-
  cowplot::align_plots(
    plot_boxplot_hatched,
    plot_glm1,
    plot_boxplot_hatched_not,
    align = c("v"),
    axis = c("lr")
  )

plot_choice_suz_mel_volume_sex <- cowplot::ggdraw() +
  cowplot::draw_plot(plot_aligned[[2]], 0, 0.15, 1, 0.67) +
  cowplot::draw_plot(plot_aligned[[1]], 0, 0.4, 1, 1) +
  cowplot::draw_plot(plot_aligned[[3]], 0,-0.37, 1, 0.98)
plot_choice_suz_mel_volume_sex
```

```
pdf("plot_choice_suz_mel_volume_sex.pdf",
    width = 9,
```

```
    height = 7)
print(plot_choice_suz_mel_volume_sex)
dev.off()
```

Mulitple plot all sex Ratio

```
plot_multi_sex <- plot_grid(plot_suzukii_reared_volume_vs_sex,
    plot_melanogaster_reared_volume_vs_sex,
    labels = c("A", "B"), label_size = 18)
plot_multi_sex
```

```
pdf("plot_multi_sex.pdf",
    width = 9,
    height = 6)
print(plot_multi_sex)
dev.off()
```
